# Supplementary material for: Community-level respiration of prokaryotic microbes may rise with global warming
Source: Nat Commun. 2019 Nov 12;10:5124. doi: 10.1038/s41467-019-13109-1 (PMC6851113; doi:10.1038/s41467-019-13109-1)
Supplement: Supplementary file 1 — Supplementary Information [file 41467_2019_13109_MOESM1_ESM.pdf]

Supporting Information for:

Community-level respiration of prokaryotic microbes may rise with global warming, Smith et. al.

Thomas P. Smith, Thomas J. H. Thomas, Bernardo García-Carreras,
Sofía Sal, Gabriel Yvon-Durocher, Thomas Bell, Samrāt Pawar

Supplementary Methods

From the information collected for each thermal response curve, we extracted data on the routine culturing conditions of each strain prior to the experiment. When organisms were provided by a cell culture bank, this temperature is simply the temperature that the strains were cultured at by the experimentalists prior to use, however in studies of novel organisms, this temperature was often also the temperature that was used during isolation of the organism. We test whether the slopes for bacteria and archaea deviate from a 1:1 line by asking whether the confidence intervals of the slopes include 1. In each case we find the slope to be significantly lower than 1.

To test whether thermal sensitivity for growth rate (fitness) reflects the thermal sensitivity of underlying metabolic fluxes, we assembled prokaryotic flux data for comparison. Metabolic flux data is sparse in the literature in comparison to growth rate data, however we were able to find some temperature-dependent flux data from a number of different studies. The various metabolic flux data that we compiled for this analysis and their respective sources are detailed in Supplementary Table 1.

In the main text we provide a simple model for changes in ecosystem respiratory carbon efflux with temperature change, based on differences in thermal sensitivity between taxa. We parameterised this for a terrestrial forest ecosystem, where the major sources of respiratory flux would be represented above-ground by plants (autotrophic eukaryotes) and below-ground by fungi (heterotrophic eukaryotes) and bacteria (heterotrophic prokaryotes). However, it is possible to extend this simple model to other ecosystem types, comprised of different taxa.

As a secondary example, we consider the short and long-term flux of marine ecosystems. Here, the major sources of biomass include heterotrophic bacteria (prokaryotes), phytoplankton (including both autotrophic eukaryotes - algae, and autotrophic prokaryotes - *Cyanobacteria*), zooplankton (heterotrophic eukaryotes) and larger animals (also heterotrophic eukaryotes)[1]. Similarly to the main text, we invoke a model based on ratios of different ecosystem components to one-another:

$$\text{Ecosystem Flux} = \delta(\beta F_{\text{hp}} + (1-\beta)F_{\text{he}}) + (1-\delta)(\gamma F_{\text{ap}} + (1-\gamma)F_{\text{ae}}). \tag{1}$$

Here, total flux is the combination of fluxes (F) produced by each component of the ecosystem (hp = heterotrophic prokaryotes, he = heteretrophic eukaryotes, ap = autotrophic prokaryotes, ae = autotrophic eukaryotes), where each flux is represented by a Boltzmann-Arrhenius function. With the proportions of each component represented by δ (overall proportion of heterotrophs to auototrophs), β (proportion of prokaryotes to eukaryotes within heterotrophic component) and γ (proportion of prokaryotes to eukaryotes within autotrophic component).

We parameterise this model using specific E values for each component and, as in the main text, calculate differences in flux with rising temperature, compared to a baseline model where all ecosystem components have the MTE derived 0.65eV E. We first parameterise this for short-term fluxes, based on $\bar{E}_S$ values with a 10°C temperature rise. For the hp component we used $E = 0.87$ ($\bar{E}_{\text{S}}$ for aerobic bacteria), for the he component we used $E = 0.65$eV ($\bar{E}_{\text{S}}$ expected under MTE, observed across many eukaryotic organisms[2]), for ap we used $E = 0.96$ ($\bar{E}_{\text{S}}$ observed for *Cyanobacteria* in our results, see main text Fig. 3) and for the ae component we used $E = 0.65$ ($\bar{E}_{\text{S}}$ for eukaryotic aquatic autotrophs is not significantly different from 0.65eV, Supplementary Figure 2). We used the same parameterisation for long-term responses, given our results linking short- and long- term thermal sensitivity in the main text ($E_G \approx \bar{E}_S$) and calculate flux differences for a 4°C temperature rise. Although we don't directly test this for autotrophic eukaryotes in the main text, previous work has shown inter-specific E in plants to be consistent with 0.65eV[3].

As with terrestrial systems, there is considerable variation in estimates for the potential biomass contributions of different marine taxa. A meta-analysis by Bar-On *et al.*[1] gives a total estimate of 1.6 Gt of carbon biomass from prokaryotes within 6 Gt total marine carbon (∼26%), see Bar-On *et al.*[1] supplementary table S23. A meta-analysis by Gasol *et al.*[4] suggests differences in biomass contributions between coastal and open ocean systems, with more heterotrophic bacterial biomass in the open ocean compared to coastal regions (∼28% heterotrophic plankton carbon in coastal systems provided by bacteria, ∼46% heterotrophic plankton carbon in oceanic systems provided by bacteria, calculated from Gasol *et al.*[4] (table 1), however this study does not assess the contribution of autotrophic bacteria. Additionally, Gasol *et al.*[4] compile estimates for the overall ratios of autotroph to heterotroph biomass in these systems, giving an average ratio of 0.98:1 heterotrophs:autotrophs in coastal systems and 1.85:1 heterotrophs:autotrophs in oceanic systems. Given these estimates we allow both β and γ to vary between 0.1 and 0.5 to provide a range of potential ecosystem biomass combinations within realistic bounds. We fix δ at 0.495 for coastal ecosystems and 0.65 for oceanic ecosystems given the ratio estimates from Gasol

et al.[4] and show a heat-map of potential flux changes for both types of marine ecosystem (Supplementary Figure 3).

In the main text we exclude *Archaea* from the ecosystem flux modelling as our data for mesophillic archaea are very restricted, leading to potentially unreliable estimates for $\bar{E}_S$ and E_G. However, were we to have reliable estimates for mesophilic, aerobic archaea, it would be trivial to also include these in such an ecosystem flux model. Extending the previous model (Supplementary Equation 1), we may invoke the following:

$$\text{Ecosystem Flux} = \delta(\beta(\epsilon F_{hb} + (1-\epsilon)F_{ha}) + (1-\beta)F_{he}) + (1-\delta)(\gamma F_{ap} + (1-\gamma)F_{ae}). \quad (2)$$

Here, heterotrophic prokaryote flux (previously F_{hp}) is broken down further into heterotrophic bacteria flux (F_{hb}) and heterotrophic archaea flux (F_{ha}), the comparative ratios of which are controlled by the new parameter, ϵ. In marine ecosystems, bacteria may overall represent in the order of ∼4 times more biomass than archaea (from the estimates given in ref.[1]), in which case we may parameterise the model with $\epsilon = 0.8$. In terrestrial ecosystems the weighting towards bacteria may be even greater[1], however there are certain more specific ecosystems where archaea are known to be more abundant such as wetlands[5].

Supplementary Discussion

We show in the main text that there is strong correlation between optimum growth temperatures, T_{pk} of prokaryotes and their evolutionary thermal regime, T_{lab} (main text Fig. 2). This may hint at rapid adaptation by these organisms to the routine culture conditions, or in the case of isolation experiments, possibly species sorting such that species isolated were simply those with the appropriate thermal tolerance range. In the high temperature range we see that in Bacterial strains T_{pk} begins to tend to fall below T_{lab}, potentially indicating that this temperature range is past the limit of thermal adaptation for Bacteria - i.e. despite being routinely cultured at high temperatures, these strains are unable to adapt to these temperatures due to the inherent biological properties of Bacterial cells. It is often stated that organisms tend to operate at temperatures below their peak temperature allowing a buffer before temperature becomes detrimental to fitness, and this is borne out in our data with T_{pk} tending to be marginally higher than T_{lab} (main text Fig. 2). It is important to note however that whilst we take these results as evidence of adaptation to culture conditions (in combination with many studies directly demonstrating this adaptive capacity[6,7,8]), this effect may also be due in part to researchers' abilities to find the best conditions to grow their bacterial strains in.

In order to further interpret the potential ecological impacts of our results, we investigated variation of thermal sensitivity within the autotroph respiration dataset. We see that whilst mean E_S may be marginally higher for aquatic autotrophs (0.67eV, CI 0.54-0.80) compared to terrestrial autotrophs (0.61eV, CI 0.53-0.69), there is a significant overlap of confidence intervals, which include 0.65eV in both cases (Supplementary Figure 2). Therefore we can not report a statistically significant difference in $\bar{E}_S$ for terrestrial and aquatic autotrophs. Differences in how organisms from different habitats respond to temperature may be driven by differences in the phylogenetic composition of those habitats. We find that the thermal sensitivity of vascular plants in our dataset is consistent with the MTE 0.65 observation (vascular plant $\bar{E}_S = 0.62$, CI 0.54-0.71) and that CIs for both green and red algae also include 0.65eV (red algae $\bar{E}_S = 0.77$, CI 0.57-0.98; green algae $\bar{E}_S = 0.50$, CI 0.33-0.68). CIs for mosses and brown algae fall significantly outside of this range (moss $\bar{E}_S = 0.92$, CI 0.78-1.03; brown algae $\bar{E}_S = 0.37$ CI 0.23 - 0.49). However, the wide confidence intervals for green and red algae and the small number of observations for mosses and brown algae suggest that our dataset is of too limited scope to draw general conclusions for these autotroph groups.

Parameterising our model for aquatic systems shows an even greater disparity between temperature induced short-term flux estimations based on a global 0.65eV activation energy versus estimations using our thermal sensitivity averages for major groups (see Supplementary Figure 3). This is due to the autotrophic component of aquatic systems also containing prokaryotes and these *Cyanobacteria* having on average even greater thermal sensitivities than heterotrophic bacteria. The accuracy of the ecosystem modelling approaches taken here and in the main text are dependant upon reliable estimates of biomass distributions in different environments. Various meta-analyses have attempted to estimate the distributions of different taxa in different ecosystems, however each has significant sources of error and give very wide ranges of possible distributions (see Bar-On *et al.*[1], Gasol *et al.*[4], del Giorgio & Gasol[9], Whitman *et al.*[10], Joergensen & Wichern[11]). Additionally the biomass distributions of heterotrophs to autotrophs have been shown to vary with warming in freshwater ecosystems[12], further complicating the parameter-

isation of these models under climate change. We have thus taken a simplistic, global approach to this modelling in order to illustrate the impacts of our findings.

Supplementary Figures

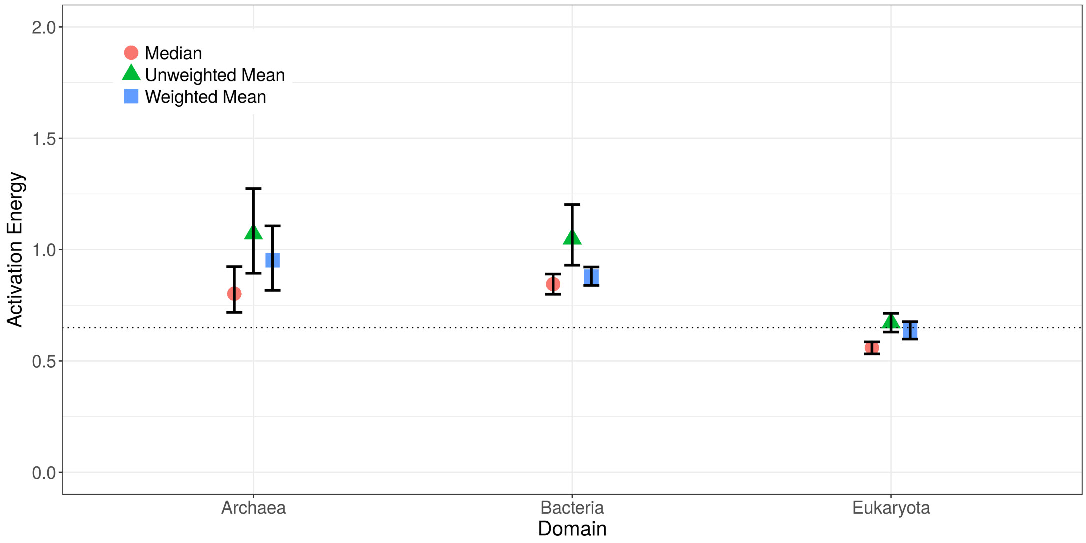

Supplementary Figure 1: Comparison of intra-specific thermal sensitivities (E_S) calculated as the median (red circles), unweighted mean (green triangles) and weighted mean (blue squares) respectively. The median is lower than the mean E_S for both Bacteria and Archaea as expected due to the right-skew of the data[2], however in all cases E_S is above the 0.65eV average (dotted line) expected from the Metabolic Theory of Ecology, which we find only holds for Eukaryotes (Main text Fig. 4). Error bars represent bootstrapped 95% CIs in each case. Source data are provided as a source data file.

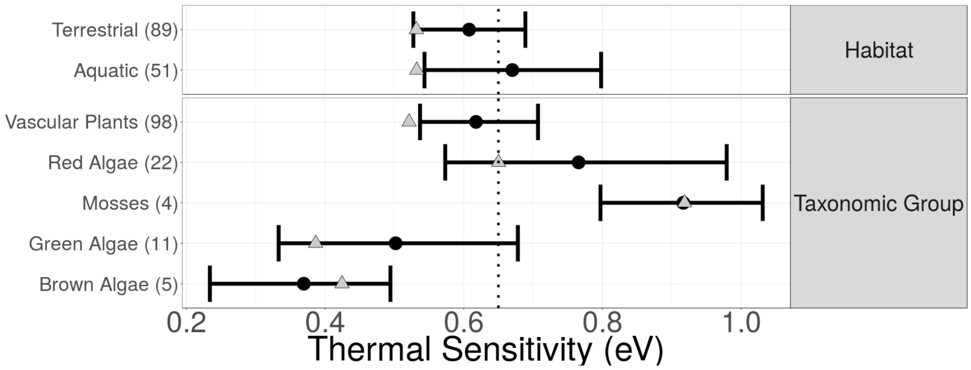

Supplementary Figure 2: Comparison of intra-specific thermal sensitivities ($\bar{E}_S$) for autotroph respiration across taxonomic and ecological divides. As in main text figure 4, points and error bars represent weighted mean and 95% CIs of E_S for each group. The number in brackets indicates the number of data points $\bar{E}_S$ was calculated from for each grouping, the dotted line marks 0.65eV and grey triangles mark the median E_S for each group. Source data are provided as a source data file.

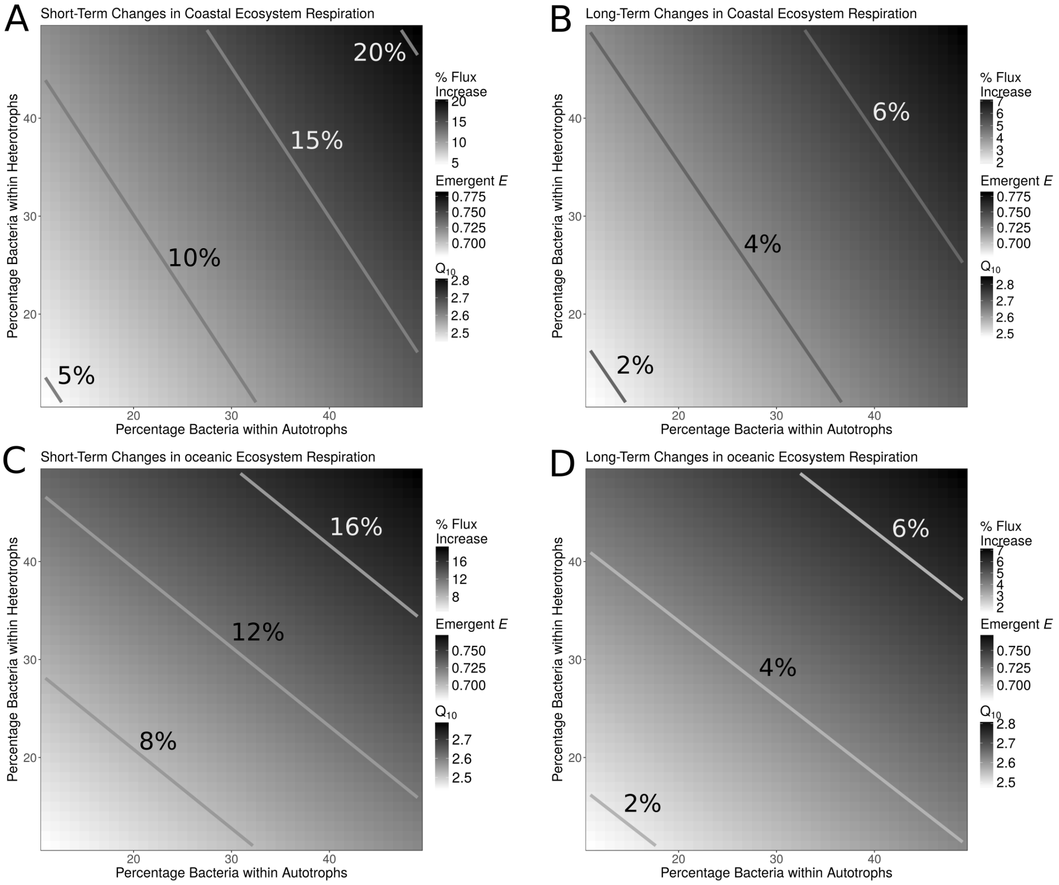

Supplementary Figure 3: **Potential changes in climate-driven aquatic ecosystem carbon flux due to differences in sensitivity between prokaryotic and eukaryotic thermal responses. A.** Heat map of % short-term increase in flux with 10°C temperature increase of model coastal ecosystems with bacteria having a different activation energy on average than eukaryotes, relative to ecosystems with all components having the same (0.65eV) average activation energy. The flux change is shown over a range of ecosystem biomass compositions in terms of bacteria vs eukaryotes within autotrophic and heterotrophic ecosystem components. The x and y axes represent γ and β respectively (in Supplementary Equation 1), expressed as percentages. The overall δ is set to 0.495 based on ref.[4]. The scale of emergent activation energies and Q_{10}s for the ecosystems with amplified flux are also shown. **B.** As A, but for long-term flux changes with a 4°C temperature increase due to climate change. **C.** and **D.** as A and B (short- and long-term) respectively, but for oceanic ecosystems with $\delta = 0.65$. As we parameterised these assuming $\bar{E}_{\mathrm{S}} \approx E_{\mathrm{G}}$, emergent ecosystem E is constant between short- and long-term plots, with only the difference in temperature change influencing the flux increase.

Supplementary Tables

Supplementary Table 1: Metabolic fluxes which we fitted TPCs to for comparison to growth rate TPCs.

Metabolic Flux	References
Sulfur oxidation rate	[13]
Fe3+ oxidation rate	[13]
Fe2+ oxidation rate	[13,14]
Sulfide production rate	[15]
Methanogenesis	[16,17,18,19,20,21,22]
Nitrate production rate	[23]
Nitrate removal rate	[24]
Fe2+ production rate	[25]
Dehalogenation rate	[26]
Sulfate reduction rate	[27,28,29]
Caffeine degradation rate	[30]
Hydrogen sulfide production rate	[31]

Supplementary References

1. Bar-On, Y. M., Phillips, R. & Milo, R. The biomass distribution on Earth. *Proceedings of the National Academy of Sciences* **115**, 6506–6511 (2018).

2. Dell, A. I., Pawar, S. & Savage, V. M. Systematic variation in the temperature dependence of physiological and ecological traits. *Proceedings of the National Academy of Sciences of the United States of America* **108**, 10591–10596 (2011).

3. Gillooly, J. F., Brown, J. H. & West, G. B. Effects of Size and Temperature on Metabolic Rate. *Science* **293**, 2248–2252 (2001).

4. Gasol, J. M., Del Giorgio, P. A. & Duarte, C. M. Biomass distribution in marine planktonic communities. *Limnol. Oceanogr* **42**, 1353–1363 (1997).

5. Stoeva, M. K. *et al.* Microbial community structure in lake and wetland sediments from a high arctic polar desert revealed by targeted transcriptomics. *PLoS ONE* **9**, 1–12 (2014).

6. Bennett, A. F., Dao, K. M. & Lenski, R. E. Rapid evolution in response to high-temperature selection. *Nature* **346**, 79–81 (1990).

7. Kishimoto, T. *et al.* Transition from positive to neutral in mutation fixation along with continuing rising fitness in thermal adaptive evolution. *PLoS Genetics* **6**, 1–10 (2010).

8. Blaby, I. K. *et al.* Experimental evolution of a facultative thermophile from a mesophilic ancestor. *Applied and Environmental Microbiology* **78**, 144–155 (2012).

9. del Giorgio, P. & Gasol, J. M. Biomass distribution in freshwater plankton communities. *The American Naturalist* **146**, 135–152 (1995).

10. Whitman, W. B., Coleman, D. C. & Wiebe, W. J. Prokaryotes: the unseen majority. *Proc. Natl. Acad. Sci. U.S.A.* **95**, 6578–6583 (1998).

11. Joergensen, R. G. & Wichern, F. Quantitative assessment of the fungal contribution to microbial tissue in soil. *Soil Biology and Biochemistry* **40**, 2977–2991 (2008).

12. Yvon-Durocher, G., Montoya, J. M., Trimmer, M. & Woodward, G. Warming alters the size spectrum and shifts the distribution of biomass in freshwater ecosystems. *Global Change Biology* **17**, 1681–1694 (2011).

13. Franzmann, P. D., Haddad, C. M., Hawkes, R. B., Robertson, W. J. & Plumb, J. J. Effects of temperature on the rates of iron and sulfur oxidation by selected bioleaching Bacteria and Archaea: Application of the Ratkowsky equation. *Minerals Engineering* **18**, 1304–1314 (2005).

14. Golyshina, O. V. *et al.* Ferroplasma acidiphilum gen. nov., sp. nov., an acidophilic, autotrophic, ferrous-iron-oxidizing, cell-wall-lacking, mesophilic member of the Ferroplasmaceae fam. nov., comprising a distinct lineage of the Archaea. *International Journal of Systematic and Evolutionary Microbiology* **50**, 997–1006 (2000).

15. Boyd, E. S. *et al.* Isolation, characterization, and ecology of sulfur-respiring Crenarchaea inhabiting acid-sulfate-chloride-containing geothermal springs in Yellowstone National Park. *Applied and Environmental Microbiology* **73**, 6669–6677 (2007).

16. Zeikus, J. G. & Wolfe, R. S. Methanobacterium thermoautotrophicus sp. n., an anaerobic, autotrophic, extreme thermophile. *Journal of Bacteriology* **109**, 707–715 (1972).

17. Zehnder, A. J. B. & Wuhrmann, K. Physiology of a Methanobacterium strain AZ. *Archives of Microbiology* **111**, 199–205 (1977).

18. Zinder, S. H. & Mah, R. A. Isolation and characterization of a thermophilic strain of Methanosarcina unable to use H2-CO2 for methanogenesis. *Applied and Environmental Microbiology* **38**, 996–1008 (1979).

19. Huser, B. A., Wuhrmann, K. & Zehnder, A. J. B. Methanothrix soehngenii gen. nov. sp. nov., a new acetotrophic non-hydrogen-oxidizing methane bacterium. *Archives of Microbiology* **132**, 1–9 (1982).

20. Ferguson, T. J. & Mah, R. A. Isolation and characterization of an H2-Oxidizing Thermophilic Methanogen. *Applied and Environmental Microbiology* **45**, 265–274 (1983).

21. Zinder, S., Anguish, T. & Cardwell, S. Effects of Temperature on Methanogenesis in a Thermophilic (58-Degrees-C) Anaerobic Digester. *Applied and Environmental Microbiology* **47**, 808–813 (1984).

22. Westermann, P., Ahring, B. K. & Mah, R. a. Temperature Compensation in Methanosarcina barkeri by Modulation of Hydrogen and Acetate Affinity. *Applied and environmental microbiology* **55**, 1262–1266 (1989).

23. Mével, G. & Prieur, D. Heterotrophic nitrification by a thermophilic Bacillus species as influenced by different culture conditions. *Canadian journal of microbiology* **46**, 465–73 (2000).

24. Oshiki, M., Shimokawa, M., Fujii, N., Satoh, H. & Okabe, S. Physiological characteristics of the anaerobic ammonium-oxidizing bacterium 'Candidatus Brocadia sinica'. *Microbiology* **157**, 1706–1713 (2011).

25. Slobodkin, A., Reysenbach, A.-l., Strutz, N., Dreier, M. & Wiegel, J. Thermoterrabacterium ferrireducens gen. nov., sp. nov., a Thermophilic Anaerobic Dissimilatory Fe (III) -Reducing Bacterium from a Continental Hot Spring. *International Journal of Systematic Bacteriology* **47**, 541–547 (1997).

26. Utkin, I., Woese, C. & Wiegel, J. Isolation and Characterization of Desulfitobacterium dehalogenans gen. nov., sp. nov., an Anaerobic Bacterium Which Reductively Dechlorinates Chlorophenolic Compounds. *International Journal of Systematic Bacteriology* **44**, 612–619 (1994).

27. Isaksen, M. F. & Jørgensen, B. O. B. Adaptation of psychrophilic and psychrotrophic sulfate-reducing bacteria to permanently cold marine environments. *Applied and Environmental Microbiology* **62**, 408–414 (1996).

28. Knoblauch, C. & Jorgensen, B. B. Effect of temperature on sulphate reduction, growth rate and growth yield in five psychrophilic sulphate-reducing bacteria from Arctic sediments. *Environmental Microbiology* **1**, 457–467 (1999).

29. Mori, K., Kim, H., Kakegawa, T. & Hanada, S. A novel lineage of sulfate-reducing microorganisms: Thermodesulfobiaceae fam. nov., Thermodesulfobium narugense, gen. nov., sp. nov., a new thermophilic isolate from a hot spring. *Extremophiles* **7**, 283–290 (2003).

30. Ibrahim, S., Shukor, M. Y., Syed, M. A., Wan Johari, W. L. & Ahmad, S. A. Characterisation and growth kinetics studies of caffeine-degrading bacterium Leifsonia sp. strain SIU. *Annals of Microbiology* **66**, 289–298 (2016).

31. Oda, K. *et al.* Production of Hydrogen Sulfide by a Moderately Thermophilic Iron-Oxidizing Bacterium Strain TI-1. *Journal of Fermentation and Bioengineering* **84**, 372–374 (1997).
