# Supplementary material for: Community-level respiration of prokaryotic microbes may rise with global warming
Source: Nat Commun. 2019 Nov 12;10:5124. doi: 10.1038/s41467-019-13109-1 (PMC6851113; doi:10.1038/s41467-019-13109-1)
Supplement: Supplementary file 3 — Reporting Summary [file 41467_2019_13109_MOESM3_ESM.pdf]

nature research

Corresponding author(s): Thomas P. Smith, Samraat Pawar

Last updated by author(s): Oct 7, 2019

Reporting Summary

Nature Research wishes to improve the reproducibility of the work that we publish. This form provides structure for consistency and transparency in reporting. For further information on Nature Research policies, see Authors & Referees and the Editorial Policy Checklist.

Statistics

For all statistical analyses, confirm that the following items are present in the figure legend, table legend, main text, or Methods section.

n/a	Confirmed	
☐	☒	The exact sample size (n) for each experimental group/condition, given as a discrete number and unit of measurement
☐	☒	A statement on whether measurements were taken from distinct samples or whether the same sample was measured repeatedly
☐	☒	The statistical test(s) used AND whether they are one- or two-sided<br>*Only common tests should be described solely by name; describe more complex techniques in the Methods section.*
☐	☒	A description of all covariates tested
☐	☒	A description of any assumptions or corrections, such as tests of normality and adjustment for multiple comparisons
☐	☒	A full description of the statistical parameters including central tendency (e.g. means) or other basic estimates (e.g. regression coefficient) AND variation (e.g. standard deviation) or associated estimates of uncertainty (e.g. confidence intervals)
☐	☒	For null hypothesis testing, the test statistic (e.g. F, t, r) with confidence intervals, effect sizes, degrees of freedom and P value noted<br>*Give P values as exact values whenever suitable.*
☒	☐	For Bayesian analysis, information on the choice of priors and Markov chain Monte Carlo settings
☒	☐	For hierarchical and complex designs, identification of the appropriate level for tests and full reporting of outcomes
☒	☐	Estimates of effect sizes (e.g. Cohen's d, Pearson's r), indicating how they were calculated

Our web collection on statistics for biologists contains articles on many of the points above.

Software and code

Policy information about availability of computer code

Data collection	Plot digitizer open source software (version 2.6.8) was used to collect thermal response data from published literature.
Data analysis	Code to fit models to thermal performance curves was written in Python (version 3.5.1). Code to analyze the results and produce figures was written in R (version 3.2.2).

For manuscripts utilizing custom algorithms or software that are central to the research but not yet described in published literature, software must be made available to editors/reviewers. We strongly encourage code deposition in a community repository (e.g. GitHub). See the Nature Research guidelines for submitting code & software for further information.

Data

Policy information about availability of data

All manuscripts must include a data availability statement. This statement should provide the following information, where applicable:

- Accession codes, unique identifiers, or web links for publicly available datasets
- A list of figures that have associated raw data
- A description of any restrictions on data availability

The source data underlying Figs 2, 3, 4 and 5 and Supplementary Figs 1 and 2 are provided as a Source Data File. All other raw data is available for download from the following git repository:
https://github.com/smithtp/hotterbetterprokaryotes

Field-specific reporting

Please select the one below that is the best fit for your research. If you are not sure, read the appropriate sections before making your selection.

☐ Life sciences ☐ Behavioural & social sciences ☒ Ecological, evolutionary & environmental sciences

For a reference copy of the document with all sections, see nature.com/documents/nr-reporting-summary-flat.pdf

Ecological, evolutionary & environmental sciences study design

All studies must disclose on these points even when the disclosure is negative.

Study description	We collected thermal performance data from the literature for bacteria and archaea and fitted a model to describe the shape of each curve. We then use the peaks of these fitted curves to fit a thermal performance model across the group of species curves. We ask whether on average thermal sensitivity of the group is equivalent to the average thermal sensitivity of species within the group - we test this by comparing bootstrapped confidence intervals. Additionally, we use confidence intervals to ask whether thermal sensitivity for growth rate is, on average, equivalent to that of metabolic rate for bacteria and archaea. We also use confidence intervals to ask whether prokaryote growth or respiration thermal sensitivity and autotroph respiration thermal sensitivity deviate from the reported 0.65eV global average (do CIs for E include 0.65?).
Research sample	An exhaustive collection of thermal performance data recorded in the published literature.
Sampling strategy	Our dataset is intended to be a full representation of all published thermal performance curve data. All studies that met our criteria (had prokaryote TPC data or autotroph respiration TPC data) were included.
Data collection	Candidate TPC data was identified through manual searches of google scholar and pubmed databases. Search terms such as 'bacteria', 'bacterium', 'archaea', 'archaeon', 'temperature', 'temperature response', 'thermal response', 'growth', 'adaptation', were used to find papers with response data particularly for growth rates. Where data was presented in tabulated format, this was taken directly from the papers. When data was presented as a figure, we used 'Plot Digitizer' software to extract the data points. The prokaryote data was compiled by Thomas P. Smith, Thomas J. H. Thomas and Gabriel Yvon-Durocher. A similar search strategy was used by Sofia Sal to extract data for respiration rate TPCs in autotrophs.
Timing and spatial scale	Data collected between December 2015 and August 2016. The spatial scale is global, i.e. the data acquisition was not constrained to data from specific locations.
Data exclusions	No data were excluded manually prior to our analysis. If our thermal modeling code was unable to converge on a fit for a specific curve, that data would be excluded from further analysis (as we could not determine thermal performance parameters). If a curve had no peak, that data was excluded from the 'hotter is better' analysis as it required fits to TPC peaks.
Reproducibility	Due to the nature of our study (a global meta-analysis), we could not repeat the analysis on other datasets.
Randomization	Sample randomization was not relevant to our study as data was not allocated into different experimental groups.
Blinding	Blinding was not possible during data acquisition, as acquisition relied upon directly searching the literature for the data. Blinding was not necessary during analysis, as the whole analysis is performed using a reproducible pipeline of code which does not analyze the data based on anything other than the mathematically derived parameters of the thermal performance curves.

Did the study involve field work? ☐ Yes ☒ No

Reporting for specific materials, systems and methods

We require information from authors about some types of materials, experimental systems and methods used in many studies. Here, indicate whether each material, system or method listed is relevant to your study. If you are not sure if a list item applies to your research, read the appropriate section before selecting a response.

Materials & experimental systems

n/a	Involved in the study	
☒	☐	Antibodies
☒	☐	Eukaryotic cell lines
☒	☐	Palaeontology
☒	☐	Animals and other organisms
☒	☐	Human research participants
☒	☐	Clinical data

Methods

n/a	Involved in the study	
☒	☐	ChIP-seq
☒	☐	Flow cytometry
☒	☐	MRI-based neuroimaging
